# Systematic Review of Sensory Processing Assessment in Alzheimer’s Disease and Related Dementias

**DOI:** 10.1002/alz.095698

**Published:** 2025-01-09

**Authors:** Clarissa Benzarti, Lauren Robinson, Celeste Roberts, MaryEllen Thompson, Justin M Barber, Katherine Snyder, Elizabeth G Hunter, Elizabeth K. Rhodus

**Affiliations:** ^1^ Nova Southeastern University, Tampa, FL USA; ^2^ University of Kentucky, Lexington, KY USA; ^3^ Eastern Kentucky University, Richmond, KY USA; ^4^ University of Kentucky College of Medicine, Lexington, KY USA; ^5^ Sanders‐Brown Center on Aging, Lexington, KY USA; ^6^ Bowling Green State University, Bowling Green, OH USA

## Abstract

**Background:**

Sensory‐based interventions are increasingly utilized in dementia care; however, evidence of effectiveness remains mixed. Assessment of sensory processing in persons with neurological impairment prior to intervention provides avenues to tailor sensory‐based intervention allowing for person‐centered care and potential for greater effectiveness. While such assessments are commonly used in pediatrics, there is limited evidence exploring application in Alzheimer’s disease and related dementias (ADRD). This systematic review evaluated evidence‐based use of sensory processing assessments in ADRD research.

**Methods:**

Following the Preferred Reporting Items for Systematic Reviews and Meta‐Analyses (PRISMA), databases (MEDLINE via PubMed, PsycINFO, CINAHL with full text, OTseeker, and Cochrane) were searched by a medical librarian for articles published in 1990‐2023. Along with pre‐specified MESH terms, inclusion criteria required articles to specify sensory processing assessment and ADRD. Independent reviewers screened titles and abstracts of all identified articles, and three reviewers conducted full text review of remaining articles. The review team held meetings to determine consensus based on inclusion/exclusion criteria.

**Results:**

The comprehensive search identified 14,928 articles of which 3,105 were removed due to duplication and out of date window. Following screening, 1,331 articles were identified for full text review leading to in‐depth review of 21 articles. After exhaustive review, no articles fully fit the inclusion criteria of assessment of sensory processing in persons with ADRD.

**Conclusion:**

Findings of this systematic review highlight the critical need for evidence‐based sensory processing assessment to support person‐centered, sensory‐based interventions in dementia care. While current use of sensory‐based interventions in dementia care may offer therapeutic benefits, it is imperative that thorough assessment of the person’s sensory processing abilities and capacity be integrated into routine practice prior to intervention application. Such assessment opens opportunity to protect and fully support individuals with neurological conditions who may experience deviated responses due to impaired sensory processing. Additional research is required to advance clinical utility in the development of sensory processing assessments and sensory‐based interventions for the ADRD population.

